# Risk factors and clinical course of hungry bone syndrome after total parathyroidectomy in dialysis patients with secondary hyperparathyroidism

**DOI:** 10.1186/s12882-016-0421-5

**Published:** 2017-01-10

**Authors:** Lo-Yi Ho, Ping-Nam Wong, Ho-Kwan Sin, Yuk-Yi Wong, Kwok-Chi Lo, Shuk-Fan Chan, Man-Wai Lo, Kin-Yee Lo, Siu-Ka Mak, Andrew Kui-Man Wong

**Affiliations:** Department of Medicine & Geriatrics, Kwong Wah Hospital, 25 Waterloo Road, Kowloon, Hong Kong SAR, China

**Keywords:** Metabolic bone disease, Dialysis, Hypocalcaemia, Parathyroidectomy, Secondary hyperparathyroidism

## Abstract

**Background:**

Hungry bone syndrome (HBS) is an important postoperative complication after parathyroidectomy for severe secondary hyperparathyroidism (SHPT). There is, however, little data in the literature on its detailed clinical course, and the associated risk factors remain controversial.

**Methods:**

We did a single-center retrospective study on 62 consecutive dialysis patients who underwent total parathyroidectomy for SHPT to examine the risk factors, clinical course and outcome. Data on demographic characteristics, perioperative laboratory parameters including serum calcium, phosphate, alkaline phosphatase (ALP) and parathyroid hormone (PTH), drug treatment for SHPT and operative details of parathyroidectomy were collected.

**Results:**

Seventeen (27.4%) patients developed severe postoperative hypocalcemia with HBS. The serum calcium dropped progressively while serum ALP rose after operation until 2 weeks later when serum calcium reached the trough and serum ALP peaked. Serum phosphate also fell but stabilized between 4 and 14 days. The total postoperative calcium and vitamin D supplementation was significantly larger, and hospital stay was significantly longer in the group with HBS as compared with those without HBS. Young age, high body weight, high preoperative ALP level, and low preoperative calcium level independently predicted the development of HBS while preoperative PTH and use of cinacalcet or paricalcitol did not.

**Conclusion:**

HBS was common after total parathyroidectomy in patients with SHPT, and it is important to closely monitor the postoperative serum calcium, phosphate and ALP levels in the following 2 weeks, especially for those at risk. The implications of our findings on perioperative management are discussed.

## Background

Secondary hyperparathyroidism (SHPT) is a common complication of chronic kidney disease (CKD), which might ultimately develop in nearly all patients with end-stage renal disease (ESRD) [1]. Despite aggressive medical therapy, parathyroidectomy continues to be necessary in those patients with severe SHPT. Hypocalcaemia is the most common medical complication following parathyroidectomy, which could be severe and prolonged in some situations, where a poorly defined entity called “hungry bone syndrome” (HBS) has been used to describe this phenomenon [2].

HBS was first described in 1948 in patients with prolonged hypocalcaemia after parathyroidectomy for primary hyperparathyroidism (HPT) [2]. Old age, size of resected parathyroid glands, preoperative serum parathyroid hormone (PTH) level, preoperative serum alkaline phosphatase (ALP) level, and serum urea nitrogen concentration were found to be risk factors [3–6]. Nevertheless, the situation in SHPT should be more complicated, to which the above findings in HPT might not be applicable.

Renal osteodystrophy in patients with ESRD encompasses a wide range of bone abnormalities including osteitis fibrosa, adynamic bone disease, osteomalacia, and mixed uremic osteodystrophy [7]. The exact bone abnormality present in individual dialysis patients, which directly affects the bone and metabolic response as well as postoperative course, is highly variable and could not be reliably predicted by serum PTH level [7, 8]. In this context, there is, indeed, little data in the literature concerning the clinical course and risk factors of severe prolonged hypocalcemia or hungry bone syndrome in ESRD patients with SHPT undergoing total parathyroidectomy. In particular, there have been some new drugs, including paricalcitol and cinacalcet, recently available for the treatment of SHPT, and it would be interesting to know whether these drugs would impact on the postoperative course and development of HBS. We therefore did a study to examine the clinical course and risk factors of HBS in a cohort of dialysis patients undergoing total parathyroidectomy. The findings could be of substantial help in the prevention of HBS and perioperative management in this group of patients.

## Methods

It was a retrospective study on 62 consecutive dialysis patients who underwent parathyroidectomy because of SHPT between January 1, 2004, and February 28, 2014, in a regional hospital with a program of about 300 prevalent peritoneal dialysis (PD) and 100 hemodialysis (HD) patients. Indications for parathyroidectomy included: severe SHPT (persistent PTH > 85 *p*mol/L) refractory to medical therapy with calcitriol, or vitamin D analogs, or calcimimetics, or a combination of calcimimetics and calcitriol or vitamin D analogs at maximal doses tolerated by individual patients; severe SHPT associated with hypercalcaemia (total calcium corrected for albumin concentration > 2.60 mmol/L) and/or hyperphosphataemia (>2.0 mmol/L) precluding further approaches with medical therapy; and calciphylaxis associated with SHPT. The study was approved by Hong Kong Hospital Authority Kowloon West Cluster Clinical Research Ethics Committee.

The medical records were reviewed and data including patient gender, age at the time of surgery, and operative-, laboratory-, medication-, and dialysis-related data were collected. The serial results of the following laboratory parameters preoperatively and, for multiple time points, postoperatively were identified: hemoglobin, serum albumin, calcium, phosphate, alkaline phosphatase (ALP), and intact parathyroid hormone (PTH) levels. Except for the early postoperative period when frequent and close monitoring of serum calcium was required, blood sampling was performed immediately before the commencement of individual dialysis session in HD patients. In addition, detailed information about preoperative medications, including daily doses of phosphate-binding drugs, active vitamin D analogs and calcimimetics were recorded. For active vitamin D analogs and calcimimetics, the exposure to such agents was counted only if the patients had taken the drug for 30 days or more within the 90-day period preceding the surgery. Cumulative doses of calcium and vitamin D supplements at 14 days, 1 month, 3 months and 12 months after surgery were calculated.

Hungry Bone Syndrome was defined as profound and prolonged hypocalcaemia with corrected serum calcium level of 2.1 mmol/L or below lasting for 4 or more days, that occurred anytime within 1 month following parathyroidectomy, despite standard postoperative supportive treatment of our unit. Patients developing HBS according to the definition were compared with those without HBS in terms of baseline characteristics, clinical course and length of hospitalization, and multivariate analysis was performed to evaluate the effect of age, sex, body weight, vintage on dialysis, Kt/V, size of the resected parathyroid glands, preoperative laboratory parameters including serum PTH, ALP, calcium, phosphate, hemoglobin and albumin as well as SHPT drug treatment for the development of HBS.

### Surgical procedure

All patients underwent bilateral neck exploration with an attempt to identify all parathyroid glands. When four or more glands were identified at the time of surgery, total parathyroidectomy was intended without doing any autotransplantation. If fewer than four glands were found, all identifiable parathyroid glands were removed.

### Perioperative treatment strategy

All patients undergoing parathyroidectomy were started on oral alfacalcidol 2 micrograms twice daily for 2 days before surgery if they were not receiving any form of active vitamin D analog before the surgery. After surgery, all patients received a normal calcium dialysis bath. All patients were started on intravenous calcium infusion at a rate of 2720 mg elemental calcium (Ca)/day (as mixture of 50 ml 10% calcium chloride solution and 450 ml normal saline solution). Serum calcium levels were monitored every 6 h starting from the time immediately after surgery. If serum calcium level was 2.1 mmol/L or below, an extra 10 ml of 10% calcium chloride solution (272 mg elemental Ca) was given. If serum calcium level was 2.4 mmol/L or above, intravenous calcium chloride infusion was stopped for 6 h and then restarted. Cinacalcet or paricalcitol therapy was stopped if present. When they were fully awake, patients were prescribed with oral calcium supplements and vitamin D analogs (alfacalcidol or calcitriol) with the dosage titrated against the serum calcium levels to allow cessation of calcium infusion. The frequency of serum calcium level monitoring and the dosage of intravenous calcium infusion were adjusted by the attending physician the next day after operation. Patients were discharged when calcium levels remained stable in the normal range.

### Statistical analysis

Statistical analyses of collected data were performed using SPSS version 21.0. Data were expressed as mean ± SD if normally distributed, or median and interquartile range otherwise. For univariate analysis, categorical variables were compared using the chi-square test or Fisher’s exact test where appropriate. Continuous variables were compared using independent samples *T*-test or Mann-Whitney *U* test for data of non-normal distribution. Variables with a *p* value of <0.05 in univariate analysis were entered into the multiple logistic regression model. Variables with *p* < 0.05 (2 tailed) were considered statistically significant.

## Results

### Baseline characteristics, surgical procedures, parathyroid pathology

There were 46 female and 16 male patients with a mean age of 52.6 ± 12.2 years (Table 1). There was no predominant primary renal diagnosis in these patients. At the time of surgery, 17 (27.4%) patients were receiving HD and 45 (72.6%) were receiving PD. Mean vintage on dialysis was 5.9 ± 2.6 years.

**Table 1:** Baseline demographics and clinical data

No. of patients	62
Gender (M:F)	16:46
Age at time of surgery (years)	52.6 ± 12.2
Body weight (kg)	52.9 (46–65)
Underlying renal diagnosis, no. of patients (%)	
Diabetic nephropathy	9 (14.5)
Hypertensive nephropathy	10 (16.1)
Polycystic kidney	4 (6.5)
Glomerulonephritis	20 (32.2)
Unknown	19 (30.6)
RRT at time of surgery	
PD	45 (72.6)
HD	17 (27.4)
Duration of dialysis (years)	5.9 ± 2.6
Kt/V (weekly)	2.31 ± 0.9
Preoperative serum biochemistry	
iPTH–peak (pmol/L)	170 (131–221)
iPTH–immediately before operation (pmol/L)	156.5 (122–215)
ALP–peak (IU/L)	309.5 (192–511)
ALP–immediately before operation (IU/L)	261 (161–447)
Calcium^a^ (mmol/L)	2.57 ± 0.21
Phosphate (mmol/L)	2.28 ± 0.65
Albumin (g/L)	31 ± 5
Haemoglobin (g/dL)	9.9 ± 1.5
Preoperative drug treatment	
Phosphate binders (*n* = 31)	
Calcium carbonate (*n* = 21) (mg/day)	2904.8 ± 1921.1
Aluminium hydroxide (*n* = 4) (mg/day)	1400.0 ± 632.5
Lanthanum (*n* = 3) (mg/day)	1083.3 ± 381.9
Sevelamer (*n* = 4) (mg/day)	2100.0 ± 1194.4
Active vitamin D (*n* = 23)	
Calcitriol (*n* = 3) (μg/week)	1.00 ± 0.50
Alfacalcidol (*n* = 12) (μg/week)	1.17 ± 0.83
Paricalcitol (*n* = 8) (μg/week)	24.00 ± 14.30
Cinacalcet (*n* = 14) (mg/day)	35.7 ± 16.2
Surgical procedure, no. of patients (%)	
4 glands resected	48 (77.4)
Less than four glands resected	14 (22.6)
Weight of resected parathyroid tissue (g)	2.56 ± 1.56
Pathology of parathyroid tissue, no. of patients (%)	
Hyperplasia	59 (95.2)
Adenoma	3 (4.8)

*Abbreviations*: *PD* Peritoneal dialysis, *HD* Hemodialysis, *iPTH* intact parathyroid hormone, *ALP* alkaline phosphatase

^a^Adjusted by serum albumin level

Within 90 days preoperatively, 31 (50%) patients received one or more phosphate binders, 23 (37.1%) received active vitamin D sterols (three had calcitriol, 12 had alfacalcidol, eight had paricalcitol), and 14 (22.6%) received calcimimetics (cinacalcet).

48 (77.4%) patients had all four glands identified and resected during surgery. The pathology revealed parathyroid hyperplasia in 95.2% of cases.

### Post-operative bone mineral metabolism and hungry bone syndrome

**Fig. 1:**
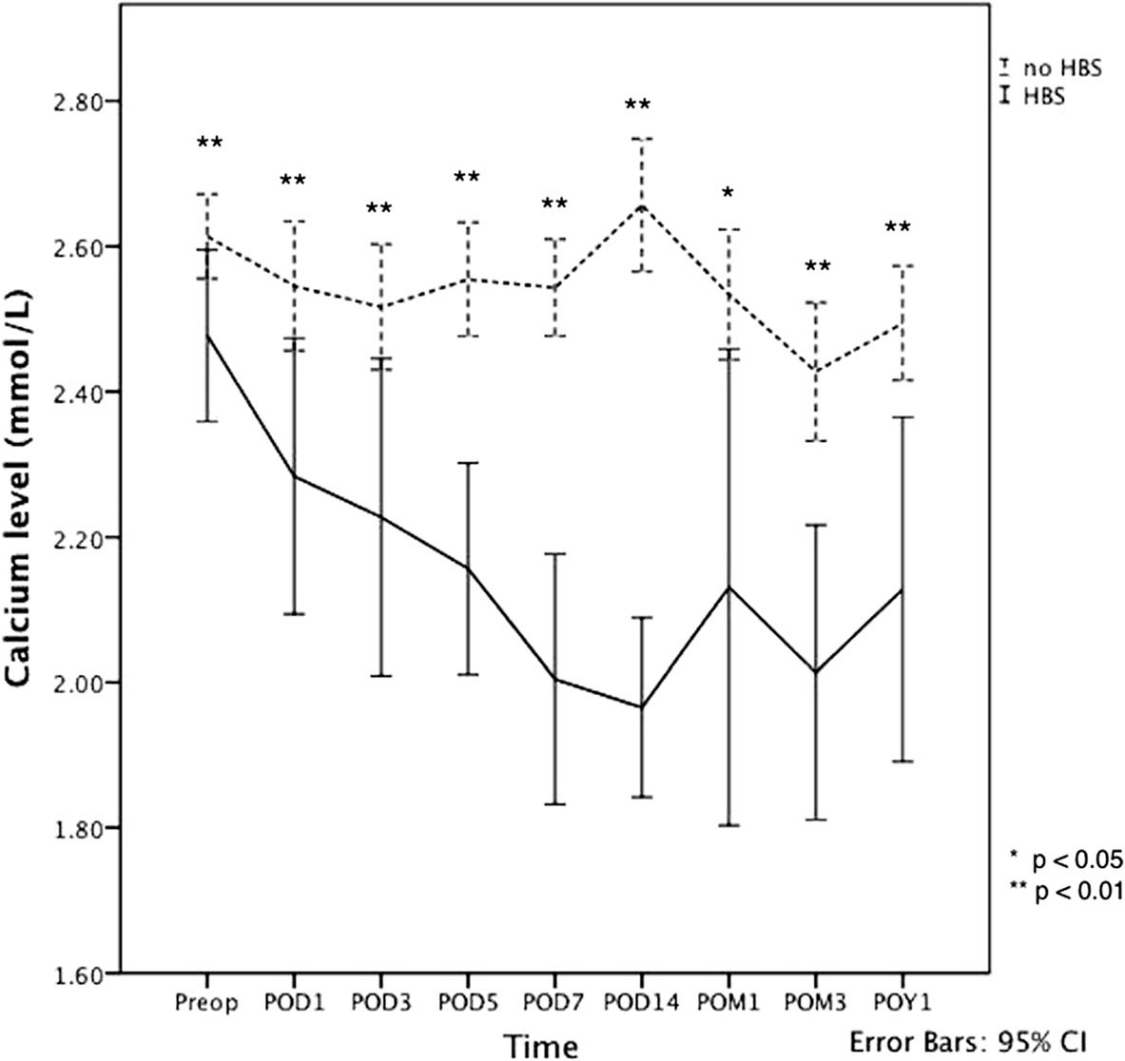
Serum calcium level before and after parathyroidectomy. Abbreviations: Preop, preoperative; POD, days of operation; POM, months after operation; POY, years after operation; HBS, hungry bone syndrome; CI, confidence interval. Solid line: patients with HBS; Dotted line; patients without HBS

The mean serum calcium level of the 62 patients decreased from 2.57 ± 0.21 mmol/L preoperatively to 2.47 ± 0.29 mmol/L on postoperative day 1 (*p* <0.0001). There were 17 patients (27.4%) who developed severe hypocalcemia with the onset of hypocalcaemia ranging from day 1 to day 14 after surgery fulfilling the diagnosis of HBS according to our study definition (Fig. 1). However, they all suffered no symptom of hypocalcaemia. While the serum calcium levels of patients without HBS appeared to stabilize after the first few days, the fall of serum calcium in patients with HBS tended to last longer with the trough occurring at about 2 weeks after the operation. The serum calcium levels of patients with HBS remained significantly lower throughout the following up period up to 1 year after operations when compared with patients without HBS.

**Fig. 2:**
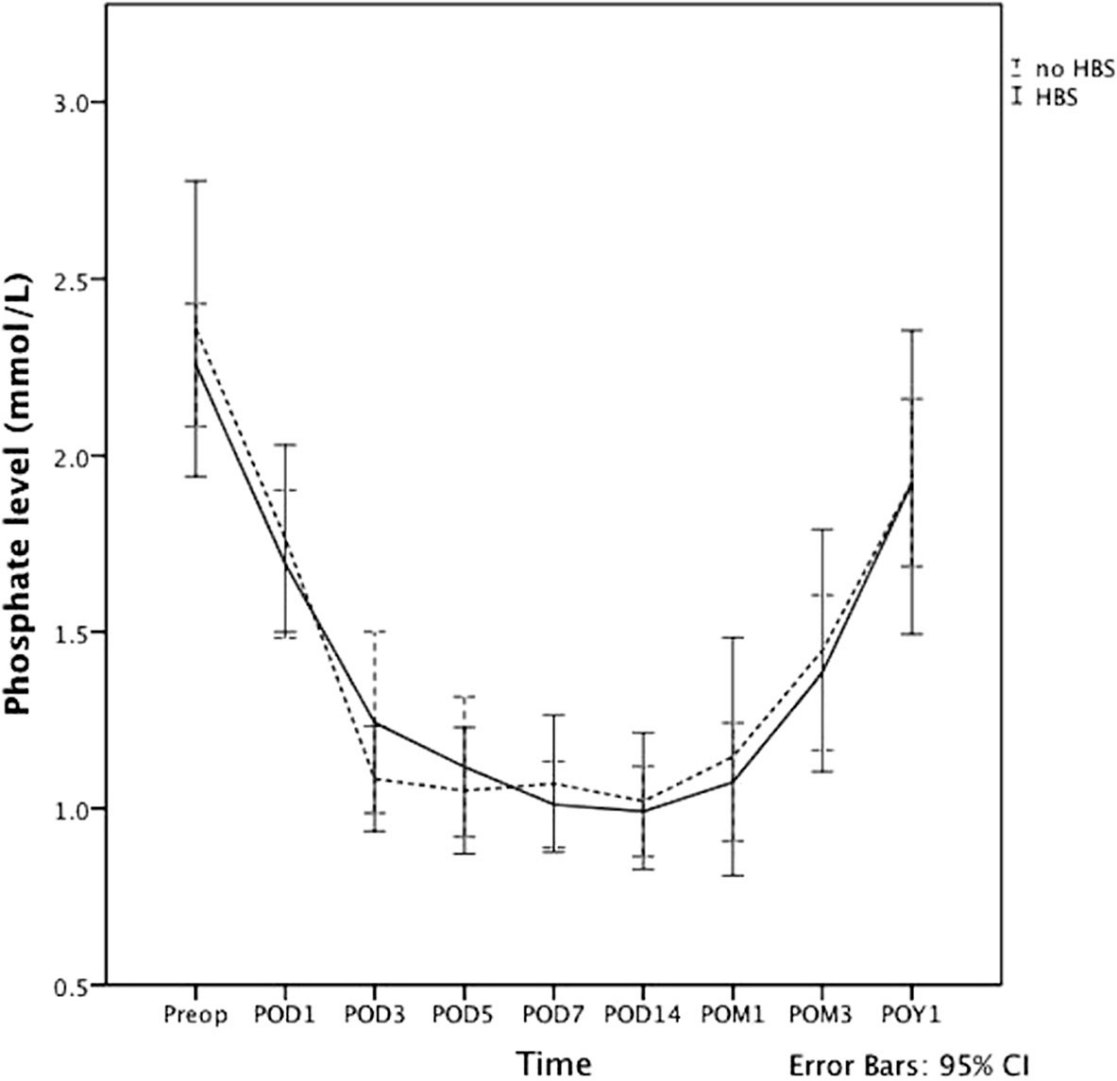
Serum phosphate level before and after parathyroidectomy. Abbreviations: Preop, preoperative; POD, days of operation; POM, months after operation; POY, years after operation; HBS, hungry bone syndrome; CI, confidence interval. Solid line: patients with HBS; Dotted line; patients without HBS

The mean serum phosphate level of the 62 patients decreased from 2.28 ± 0.65 mmol/L preoperatively to 1.75 ± 0.54 mmol/L on postoperative day 1 (*p* < 0.0001). The serum phosphate level tended to stabilize between 4 and 14 days after the operation. Hypophosphataemia with serum phosphate level of < 0.8 mmol/L was observed in 24 (38.7%) patients within 1 month following parathyroidectomy. There was no significant difference in the serum phosphate level between patients with and without HBS. The change in serum phosphate level over time was shown in Fig. 2.

**Fig. 3:**
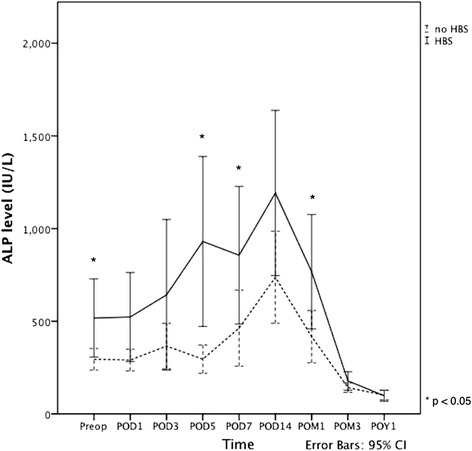
Serum ALP level before and after parathyroidectomy. Abbreviations: Preop, preoperative; POD, days of operation; POM, months after operation; POY, years after operation; HBS, hungry bone syndrome; ALP, alkaline phosphatase; CI, confidence interval. Solid line: patients with HBS; Dotted line; patients without HBS

Generally, serum ALP level increased progressively after surgery in both groups, attaining their peak levels at 2 weeks postoperatively and then decreased gradually (Fig. 3). By 3 months, 54.8% of all patients had their ALP level fall within the normal limit, and 1 year after surgery 88.7% of them had the ALP level normalized. In addition to having a higher preoperative level, patients with HBS also showed a significantly higher serum ALP level at day 5, day 7 and 1 month after the operation as compared with patients without HBS.

77.4% of the patients had their iPTH levels normalized after surgery within 1 week’s time. At 1-year follow-up, 12 (19.4%) patients had residual/ recurrent hyperparathyroidism as evidenced by PTH levels persistently above the normal reference range.

### Comparison between patients with or without HBS

#### Calcium and vitamin D supplementation

Table 2 summarized the results on postoperative use of calcium and vitamin D supplementation in the 62 patients divided into the two outcome groups. Average daily doses of calcium supplementation were significantly higher in the group with HBS compared to the group without HBS up to 1 year postoperatively. Similarly, average daily doses of vitamin D supplementation postoperatively were higher in HBS group as compared with the group without HBS throughout the study period.

**Table 2:** Calcium and active vitamin D supplementation following total parathyroidectomy

	No HBS	HBS	*p* value
Average daily dose of active vitamin D^a^ supplementation (μg)			
Postop Day 14	2.04 ± 1.46	2.89 ± 1.96	0.068
Postop Month 1	1.77 ± 1.15	3.20 ± 2.11	0.016
Postop Month 3	1.24 ± 0.78	2.87 ± 2.13	0.006
Postop Year 1	0.65 ± 0.44	2.40 ± 2.13	0.018
Average daily dose of elemental calcium^b^ supplementation (mg)			
Postop Day 14	4362.5 ± 3492.8	8551.3 ± 4671.2	<0.001
Postop Month 1	3869.2 ± 3086.0	9119.3 ± 4904.7	<0.001
Postop Month 3	2554.9 ± 1780.3	7896.9 ± 5230.1	0.001
Postop Year 1	1503.5 ± 923.2	5545.7 ± 4642.9	0.005
Hospital stay (days)	10.2 ± 2.3	15.4 ± 6.6	0.007

^a^Calcitriol or alfacalcidol

^b^In the form of oral calcium carbonate tablets (40% elemental calcium)

For patients who received active vitamin D analogs preoperatively, the requirement of calcium and vitamin D supplementation after surgery tended to be lower than those who did not receive active vitamin D analogs, but the difference did not reach statistical significance (Table 3a).

**Table 3:** Calcium and active vitamin D supplementation following total parathyroidectomy

	No preoperative use of active vitamin D	Preoperative use of active vitamin D	*p* value
a. Average daily dose of active vitamin D^a^ supplementation (μg)			
Postop Day 14	2.33 ± 1.74	2.18 ± 1.50	0.729
Postop Month 1	2.19 ± 1.68	2.12 ± 1.47	0.876
Postop Month 3	1.73 ± 1.56	1.61 ± 1.34	0.760
Postop Year 1	1.20 ± 1.71	0.91 ± 1.00	0.416
Average daily dose of elemental calcium^b^ supplementation (mg)			
Postop Day 14	6070.3 ± 4588.2	4562.7 ± 3790.0	0.179
Postop Month 1	5760.1 ± 4607.1	4543.5 ± 3790.4	0.289
Postop Month 3	4455.6 ± 4040.3	3280.5 ± 3624.0	0.255
Postop Year 1	2635.2 ± 2591.4	2328.4 ± 3733.1	0.713
	No preoperative use of cinacalcet	Preoperative use of cinacalcet	
b. Average daily dose of active vitamin D^a^ supplementation (μg)			
Postop Day 14	2.14 ± 1.61	2.73 ± 1.74	0.238
Postop Month 1	2.03 ± 1.55	2.64 ± 1.69	0.211
Postop Month 3	1.58 ± 1.43	2.03 ± 1.62	0.320
Postop Year 1	0.96 ± 1.11	1.64 ± 2.54	0.387
Average daily dose of elemental calcium^b^ supplementation (mg)			
Postop Day 14	5421.4 ± 4243.3	5818.4 ± 4424.1	0.761
Postop Month 1	5180.7 ± 4263.0	5748.0 ± 4693.8	0.670
Postop Month 3	3864.0 ± 3413.5	4553.7 ± 5381.1	0.565
Postop Year 1	2270.6 ± 1909.5	3551.9 ± 5541.6	0.446

^a^Calcitriol or alfacalcidol

^b^In the form of oral calcium carbonate tablets (40% elemental calcium)

For patients who received cinacalcet preoperatively, the requirement of calcium and vitamin D supplementation after surgery appeared to be higher than those who did not receive cinacalcet, but the difference did not reach statistical significance (Table 3b).

#### Duration of hospitalization

Length of hospital stay was significant longer in HBS group compared with the group without HBS (15.4 ± 6.6 days *vs* 10.2 ± 2.3 days, *p* = 0.007) (Table 2).

#### Risk factors

Compared with patients without HBS, patients with HBS were of younger age (47.5 years *vs* 54.5 years, *p* = 0.043), higher body weight (60.7 kg *vs* 49.8 kg, *p* = 0.01), higher preoperative serum ALP level (415 IU/L *vs* 221 IU/L, *p* = 0.008), and lower preoperative serum calcium level (2.44 mmol/L *vs* 2.60 mmol/L, *p* = 0.001) (Table 4). The proportion of male patients was also higher in HBS group compared with the group without HBS (47.1 *vs* 16.7%, *p* = 0.019). Preoperative use of active vitamin D analogs had no significant effect on the development of HBS. The proportion of patients with preoperative use of cinacalcet appeared to be higher in HBS group compared with the group without HBS but the difference did not reach statistical significance (35.3 *vs* 17.8%, *p* = 0.141).

**Table 4:** Clinical characteristics of patients with and without HBS following total parathyroidectomy

	No HBS	HBS	*p* value
No. of patients	45	17	-
Male gender	8 (16.7%)	8 (47.1%)	0.019
Age (years)	54.5 ± 12.5	47.5 ± 10.2	0.043
Body weight (kg)	49.8 (44.9–61.6)	60.7 (50.4–71.2)	0.01
PD (%)	77.8	58.8	0.136
Duration of dialysis (years)	5.9 ± 2.3	6.0 ± 3.3	0.904
Kt/V (weekly)	2.26 ± 0.80	2.47 ± 1.21	0.439
Preoperative serum biochemistry			
iPTH–peak (pmol/L)	164 (123–213)	189 (151–243)	0.144
iPTH–immediately before operation (pmol/L)	154 (117–211)	183 (137–221)	0.237
ALP–peak (IU/L)	250 (178–496)	415 (226–738)	0.051
ALP–immediately before operation (IU/L)	221 (149–397)	415 (221–727)	0.008
Calcium^a^ (mmol/L)	2.62 ± 0.18	2.44 ± 0.23	0.001
Phosphate (mmol/L)	2.26 ± 0.58	2.34 ± 0.81	0.637
Albumin (g/L)	30 ± 5	32 ± 5	0.220
Haemoglobin (g/dL)	10.0 ± 1.4	9.5 ± 1.7	0.242
Preoperative drug treatment			
Use of phosphate binders	21 (46.7%)	10 (58.8%)	0.393
Calcium carbonate	15 (33.3%)	6 (35.3%)	0.884
Aluminium hydroxide	1 (2.2%)	3 (17.6%)	0.059
Lanthanum	2 (4.4%)	1 (5.9%)	1.000
Sevelamer	4 (8.9%)	0	0.568
Use of active vitamin D	16 (35.6%)	7 (41.2%)	0.683
Calcitriol	1 (2.2%)	2 (11.8%)	0.180
Alfacalcidol	9 (20%)	3 (17.6%)	1.000
Paricalcitol	6 (13.3%)	2 (11.8%)	1.000
Use of cinacalcet	8 (17.8%)	6 (35.3%)	0.141
4 glands resected	35 (77.8%)	13 (76.5%)	0.913
Weight of resected parathyroid tissue (g)	2.42 ± 1.63	2.93 ± 1.32	0.254
Parathyroid hyperplasia	43 (95.6%)	16 (94.1%)	1.000
iPTH at postoperative week 1 (pmol/L)	2.1 (0.5–6.6)	0.7 (0.4–3.9)	0.174

*Abbreviations*: *PD* Peritoneal dialysis, *iPTH* Intact parathyroid hormone, *ALP* Alkaline phosphatase

^a^ Adjusted by serum albumin level

With the significant findings in univariate analysis, factors including gender, age, body weight, serum preoperative ALP and serum preoperative calcium levels were further evaluated using multivariate logistic regression analysis, and it showed that that young age, high body weight, high preoperative serum ALP level, and low preoperative serum calcium level independently predicted the development of HBS (Table 5). Additional analyses forcing preoperative iPTH levels into the model yielded no change in the results.

**Table 5:** Multiple Logistic Regression Model of risk factors for HBS

Variable	Unit	Relative Risk	95% Confidence Interval	*P* value
Body Weight	1 kg	1.06	1.007–1.12	0.027
Preoperative serum calcium	1 mmol/L	0.005	0.0001–0.332	0.013
Age	1 year	0.923	0.86–0.99	0.035
Preoperative ALP	1 IU/L	1.004	1.0–1.007	0.044

*Abbreviations*: *ALP* Alkaline phosphatase, *HBS* Hungry bone syndrome

## Discussion

Although there are a few retrospective studies in the literature examining the problem of post-operative hypocalcemia in dialysis patients undergoing parathyroidectomy, there was marked heterogeneity in the case definition and reported incidence ranging from 27.8 to 72%. Most studies merely focused on individual aspect such as the occurrence of early hypocalcemia immediately after the operation whereas other studies addressed the problem from other perspectives such as the length of hospital stay after operation, hospital readmission and total calcium requirement [9–13]. Since these studies just concentrated on a particular area in the postoperative course, the data might not be able to reflect the whole situation. In addition, parathyroid procedures were rather variable in these studies. The majority underwent subtotal parathyroidectomy or total parathyroidectomy with auto-transplantation while total parathyroidectomy merely accounted for a minority. Indeed, there has not been any study that could clearly delineate the postoperative course in patients with total parathyroidectomy for SHPT.

In this context, this study examined multiple aspects related to the postoperative care in patients suffering from SHPT undergoing total parathyroidectomy without autotransplantation. They included serial changes in serum calcium, phosphate and alkaline phosphatase levels, calcium and active vitamin D requirement and length of hospitalization in addition to risk factor identification for postoperative occurrence of HBS. Indeed, this study showed that in patients undergoing total parathyroidectomy, although the serum calcium level and phosphate level tended to fall immediately after the operation, there might be a delay up to 2 weeks after the operation before it reached its lowest level. At the same time, serum ALP level also increased progressively after the operation, reflecting the state of increased bone formation [14, 15], and peaked at week 2. These findings should carry important implications. First, the severity of hypocalcemia and demand of calcium replacement resulting from accelerated bone formation might not be fully reflected by the serum calcium level early after operation. In other words, patients might develop severe hypocalcemia in the later time despite a modest drop in serum calcium early postoperatively. Therefore, patients undergoing total parathyroidectomy should have their serum calcium and phosphate levels closely monitored in the following 2 weeks to safeguard against the development of severe hypocalcemia and to provide guidance on the intensity of calcium supplementation. At the same time, it appeared that the rise of serum alkaline phosphate correlated well with the decline in serum calcium and probably increasing demand of calcium replacement. Serum ALP level might therefore serve as a biomarker indicating the intensity of bone formation and the likely calcium requirement of individual patients. In practical terms, a rising serum alkaline phosphatase might portend an increasing demand for calcium supplementation and should prompt for a dose escalation whereas a declining serum ALP level would warrant consideration of dose tapering in active vitamin D and calcium to avoid overzealous replacement and inadvertent hypercalcemia.

Second, this postoperative calcium dynamic with delayed drop in serum calcium level also challenges the validity of those case definition adopted by some previous studies in examining the problem, which solely focused on serum calcium level immediately after operation. In contrast, by including all those cases having profound and prolonged hypocalcaemia lasting for 4 days or more that occurred anytime within 1 month following parathyroidectomy, it is highly unlikely that any case of significant postoperative hypocalcemia would have been missed and misclassified in this study. In other words, we believe that our definition of HBS in this study should be appropriate which could allow us to identify a particular group of patients who would likely require intensive monitoring and aggressive calcium supplementation. It follows that the risk factors found in this study should also be highly relevant and be of help in accurately identifying those high risk individuals.

With our case definition, hungry bone syndrome (HBS) was found to be present in 27.4% of cases in this study. The risk factors predisposing to its development were young age, high body weight, high preoperative ALP level, and low preoperative serum calcium level. Young age and low preoperative calcium have been reported in previous individual case series which employed various different case definitions [9–11]. The role of preoperative serum ALP level, however, remained controversial. In a case series, high preoperative ALP was shown to be predictive of hypocalcemic problem as being represented by a prolonged hospital stay, whereas protective effect was found in another study which focused on early hypocalcemia within 24 h after operation [9, 13]. On the other hand, heavy body weight was, for the first time, being identified as a risk factor. While the exact underlying mechanism remains unknown, it is plausible that patients with high body weight might have a higher bone mass and hence higher total calcium deficit [16, 17].

Preoperative PTH level was not shown to be a risk factor in the current study. There are a few possible explanations for this. First, uraemic state is associated with resistance of bone cells to the actions of PTH [18–20], and the relationship between serum iPTH levels and degree of bone remodeling is not always maintained. Second, iPTH assay not only measures the level of biologically active intact PTH (1-84), but also shows cross-reactivity with some smaller N-terminally truncated PTH fragments which have bone action opposite to intact PTH (1-84) [18–20].

No significant difference was observed between patients with or without receiving active vitamin D sterols such as paricalcitol or cinacalcet preoperatively in terms of the risk of HBS and postoperative calcium requirement. This could either be due to our small sample size or be ascribed to an indirect effect of these drugs on bone healing instead of any direct effect on HBS. Nevertheless, with medications such as paricalcitol shown to have significant effect in suppressing serum ALP in the literature, it is possible that intensive treatment with this type of agents before operation might help mitigate the risk of severe postoperative hypocalcemia [21]. On the other hand, with low serum calcium being a risk factor for HBS, overzealous treatment with cinacalcet preoperatively should probably be avoided.

The present study had several limitations. First, it was retrospective and observational in nature. Thus, the results were subject to possible selection bias and limited by suboptimal data collection. Second, the sample size was relatively small and potential significant associations among variables could have been masked. A formal multicenter registry would provide more accurate estimate of epidemiology and better understanding of clinical features of HBS following parathyroidectomy in renal SHPT in our region. Third, due to the heterogeneous definitions of significant postoperative hypocalcemia or HBS, one must be cautious when comparing the results of the present study to other similar studies.

In addition, patients receiving PD and intermittent HD were pooled into a single study population in this study, where pre-dialysis serum concentrations of individual parameters in HD patients were compared with their PD counterparts being obtained by random blood sampling. With inherent fluctuations in serum concentrations, there might be a concern that pre-dialysis serum concentrations of dialyzable uremic retention molecules, in particular, phosphorus in patient on intermittent HD might not truly reflect the overall exposure, which could potentially affect the validity of comparison. For example, the time-averaged serum concentrations of phosphorus in HD patients were significantly lower despite having the pre-dialysis concentrations comparable with midmorning concentrations of PD [22, 23]. Nevertheless, with similar proportions of HD and comparable preoperative serum phosphate levels in patients with or without HBS in this study, we believe that it should not impact significantly on the results and our univariate and multivariate analyses in risk factor identification should remain valid.

## Conclusion

HBS could be common after total parathyroidectomy. Serum calcium tended to fall soon after a successful total parathyroidectomy but the occurrence of severe hypocalcemia could be delayed up to 2 weeks after the operation. Patients should have their serum calcium, phosphate and ALP levels closely monitored in the following 2 weeks, especially for those at risk. Hopefully, with a better understanding of postoperative course and improvement of perioperative management, the occurrence of severe hypocalcemia could be avoided and any adverse consequence could be minimized.

## Abbreviations

ALP: Alkaline phosphatase
Ca: Calcium
CKD: Chronic kidney disease
ESRD: End-stage renal disease
HBS: Hungry bone syndrome
HD: Hemodialysis
iPTH: intact parathyroid hormone
PD: Peritoneal dialysis
PTH: Parathyroid hormone
SHPT: Secondary hyperparathyroidism

## References

[1] Jofre R, Lopez Gomez JM, Menarguez J (2003). Parathyroidectomy: whom and when?. Kidney Int Suppl.

[2] Albright F, Reifenstein EC (1948). The parathyroid glands and metabolic bone disease: selected studies.

[3] Brasier AR, Nussbaum SR (1988). Hungry bone syndrome: clinical and biochemical predictors of its occurrence after parathyroid surgery. Am J Med.

[4] Mittendorf EA, Merlino JI, Mc Henry CR (2004). Post-parathyroidectomy hypocalcemia: incidence, risk factors, and management. Am Surg.

[5] Kale N, Basaklar A, Sonme K, Uluoglu O, Demirsoy S (1992). Hungry bone syndrome in a child following parathyroid surgery. J Pediatr Surg.

[6] Zamboni W, Folse R (1986). Adenoma weight: a predictor of transient hypocalcaemia after parathyroidectomy. Am J Surg.

[7] KDIGO clinical practice guidelines for the diagnosis, evaluation, prevention, and treatment of chronic kidney disease-mineral and bone disorder (CKD-MBD). Kidney Int. 2009; 76(suppl 113):S1–130. 10.1038/ki.2009.188 19644521

[8] Ferreira MA (2000). Diagnosis of renal osteodystrophy: when and how to use biochemical markers and non-invasive methods; when bone biopsy is needed. Nephrol Dial Transplant.

[9] Torer N, Torun D, Micozkadioglu T (2009). Predictors of early postoperative hypocalcaemia in haemodialysis patients with secondary hyperparathyroidism. Transplant Proc.

[10] Viaene L, Evenepoel P, Bammens B, Claes K, Kuypers D, Vanrenterghem Y (2008). Calcium requirements after parathyroidectomy in patients with refractory secondary hyperparathyroidism. Nephron Clin Pract.

[11] Latus J, Roesel M, Fritz P, Braun N, Ulmer C, Steurer W, Biegger D, Alscher MD, Kimmel M (2013). Incidence of and risk factors for hungry bone syndrome in 84 patients with secondary hyperparathyroidism. Int J Nephrol Renov Dis.

[12] Goldfarb M, Gondek SS, Lim SM, Farra JC, Nose V, Lew JI (2012). Postoperative hungry bone syndrome in patients with secondary hyperparathyroidism of renal origin. World J Surg.

[13] Cheng SP, Liu CL, Chen HH, Lee JJ, Liu TP, Yang TL (2009). Prolonged hospital stay after parathyroidectomy for secondary hyperparathyroidism. World J Surg.

[14] Lu KC, Ma WY, Yu JC, Wu CC, Chu PL (2012). Bone turnover makers predict changes in bone mineral density after parathyroidectomy in patients with renal hyperparathyroidism. Clin Endocrinol.

[15] Urena P, Vernejoul MC (1999). Circulating biochemical markers of bone remodeling in uraemic patients. Kidney Int.

[16] Felson DT, Zhang Y, Hannan MT, Anderson JJ (1993). Effects of weight and body mass index on bone mineral density in men and women: the Framingham study. J Bone Miner Res.

[17] Shapses SA, Cifuentes M, Holick MF, Dawson-Hughes B (2004). Body weight/composition and weight change: effects on bone health. Nutrition and bone health.

[18] Quarles LD, Lobaugh B, Murphy G (1992). Intact parathyroid hormone overestimates the presence and severity of parathyroid-mediated osseous abnormalities in uraemia. J Clin Endocrinol Metab.

[19] Goodman WG, Ramirez JA, Belin TR (1994). Development of adynamic bone in patients with secondary hyperparathyroidism after intermittent calcitriol therapy. Kidney Int.

[20] Nguyen-Yamamoto L, Rousseau L, Brossard JH, Lepage R, D’ Amour P (2001). Synthetic carboxyl-terminal fragments of PTH decrease ionized calcium concentration in rats by acting on a receptor different from the PTH/ PTH-related peptide receptor. Endocrinology.

[21] Cozzolino M, Ketteler M, Martin KJ, Sharma A, Goldsmith D, Khan S (2014). Paricalcitol- or cinacalcet-centred therapy affects markers of bone mineral disease in patients with secondary hyperparathyroidism receiving haemodialysis: results of the IMPACT-SHPT study. Nephrol Dial Transplant.

[22] Viaene L, Meijers B, Vanrenterghem Y, Evenepoel P (2012). Daytime rhythm and treatment-related fluctuations of serum phosphorus concentration in dialysis patients. Am J Nephrol.

[23] Evenepoel P, Meijers BK, Bammens B, Viaene L, Claes K, Sprangers B, Naesens M, Hoekstra T, Schlieper G, Vanderschueren D, Kuypers D (2016). Phosphorus metabolism in peritoneal dialysis– and haemodialysis-treated patients. Nephrol Dial Transplant.

